# Effects of sevoflurane exposure during different stages of pregnancy on the brain development of rat offspring

**DOI:** 10.1007/s00540-021-02972-2

**Published:** 2021-07-19

**Authors:** Feng-he Cui, Jie Li, Ke-zhong Li, Yong-gang Xie, Xiao-ling Zhao

**Affiliations:** Department of Anesthesiology, The Affiliated Yantai Yuhuangding Hospital of Qingdao University, No. 20 Yuhuangdingdong Road, Zhifu District, Yantai, 264000 Shandong China

**Keywords:** Sevoflurane, Pregnancy, Offspring, NR4A1/NF-κB pathway

## Abstract

**Objective:**

This study explored the effects of sevoflurane exposure during different stages of pregnancy on the brain development of offspring.

**Methods:**

Thirty-six pregnant SD rats were randomly divided into 4 groups: control, sevoflurane exposure in early (S1) pregnancy, sevoflurane exposure in middle (S2) pregnancy, and sevoflurane exposure in late (S3) pregnancy. After natural birth, the learning and memory capacity of offspring rats was analyzed using the Morris water maze experiment. The hippocampi of offspring rats were collected. The levels of interleukin (IL)-1β, IL-6, and tumor necrosis factor (TNF)-α in the hippocampus were measured by ELISA. Additionally, the Nissl bodies in the hippocampus were analyzed using Nissl staining. Immunohistochemistry was used to examine the expression of BDNF and CPEB2 in the hippocampus of offspring. Proteins related to the NR4A1/NF-κB pathway were analyzed using western blotting.

**Results:**

The memory and learning capacity of offspring rats was significantly reduced in the S1 and S2 groups compared to the control group (*p* < 0.05), while there was no obvious difference between the control and S3 groups (*p* > 0.05). The level of IL-1β was significantly increased (*p* < 0.05) in the S1 group compared with the control group. Sevoflurane anesthesia received in early and middle pregnancy could significantly affect the formation of Nissl bodies in the hippocampi of offspring rats. In addition, the expression of BDNF and CPEB2 in the hippocampi of offspring rats was greatly decreased in the S1 group compared with the control group (*p* < 0.05). The expression of NR4A1 in the hippocampi of rat offspring was significantly decreased in the S1 and S2 groups compared with the control group (*p* < 0.05). The expression of proteins related to the NF-κB pathway was increased in the S1 group compared to the control group (*p* < 0.05).

**Conclusions:**

The neurotoxic effect of maternal sevoflurane anesthesia on the brain development of offspring is higher when the exposure occurs in early pregnancy than in late pregnancy, and its mechanism might involve the NR4A1/NF-κB pathway to increase the secretion of inflammatory cytokines.

## Introduction

Gestation is a crucial time in the process of brain development, and it is also the period most easily affected by external factors. The influence of prenatal factors on fetal brain development has always been a research topic of great interest. Non-obstetric surgery is needed in 0.75–2% of pregnant women, and the safety of anesthesia for mothers and children is a key consideration at this time [1]. Anesthesia is an approach for eliminating pain and creating good surgical conditions during surgery or diagnostic examination. Among the anesthetics in current use, propofol, remifentanil, and sevoflurane have been widely applied in clinical practice [2–4]. At present, the effectiveness and relative safety of sevoflurane in surgical anesthesia are recognized [5]. Sevoflurane is also one of the agents adopted for maintenance anesthesia during surgery on pregnant women [6, 7]. Clinical researchers are interested in further studying the effects of sevoflurane in different patient populations and organ systems.

A wide range of studies have suggested the potential impacts of gestational exposure to inhalational anesthetics on behavioral outcomes in offspring. Early studies by a few scholars suggested that anesthesia could not affect fetal neurodevelopment. For example, McClaine et al. administered anesthetics at an approximately clinical dose to sheep approximately 122 days into pregnancy and found no neurotoxicity through histological evaluation [8]. However, a new study in 2020 reported that desflurane anesthesia during pregnancy might harm the learning and memory functions of juvenile offspring; mice displayed increased sensitivity to fear conditioning if their mothers were administered 10% desflurane for 3 h while gestating them [9]. Another study showed that the inhalational anesthetic sevoflurane could act on the fetus through the placenta and that high-dose, long-term exposure posed the highest degree of neurotoxicity risk to fetal and juvenile mice [10]. Multiple animal studies have investigated the effects of gestational sevoflurane anesthesia on offspring in the neonatal period. Sevoflurane anesthesia damages the nervous system of the offspring, and this damage is associated with apoptosis [11], oxidative stress [12], inflammation [13], iron deficiency [14], and other changes. However, these studies focused on sevoflurane exposure during only one stage of pregnancy. Therefore, the effects of maternal sevoflurane exposure on offspring rats during different stages of pregnancy need to be studied, and the related mechanisms also need to be explored further.

In this study, sevoflurane exposure was administered to rats at different stages of pregnancy, and then the memory and learning capacity of the offspring rats was analyzed. The effects of sevoflurane exposure on the hippocampi of offspring rats were analyzed to preliminarily explore the possible mechanism.

## Materials and methods

### Experimental animals

Thirty-six specific pathogen free grade pregnant Sprague–Dawley rats (3–5 days of gestation, 250 ± 30 g) were purchased from Jinan Pengyue Experimental Animal Breeding Co., Ltd. (Jinan, Shandong, China, SCXK (Lu) 20190003). The rats were kept in standard housing conditions, which consisted of a temperature range of 23 ± 2 °C, an average humidity of 55 ± 5%, a 12 h–12 h light–dark cycle, and free access to food and water. The animal experiments were reviewed and approved by the Animal Care and Use Committee of the Affiliated Yantai Yuhuangding Hospital of Qingdao University.

### Grouping and treatment of animals

A random number table was used to divide pregnant rats into a control group (Control) and three groups that underwent sevoflurane inhalation in early, middle, and late pregnancy (S1, S2, and S3, respectively). Each group contained 9 pregnant rats.

**Fig. 1:**
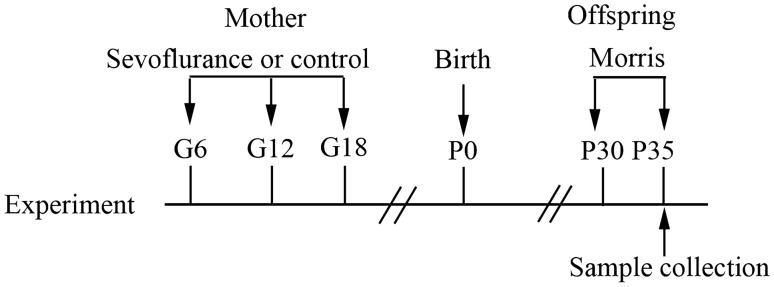
Experimental process

The pregnant rats in the control group received 100% oxygen without sevoflurane for 2 h. The rats in the S1 group were anesthetized with 2% sevoflurane for 2 h on the 6th day of pregnancy. The rats in the S2 group were anesthetized with the same dosage on the 12th day of pregnancy, and the rats in the S3 group were anesthetized similarly on the 18th day of pregnancy [15]. During the experiment, 100% oxygen was used as the carrier gas, and the total gas flow rate was 2 L/min. After the pregnant rats awoke naturally, they were returned to their cages to continue feeding and give birth naturally. The experimental process is shown in Fig. 1.

### Sample collection

On the 30th day after the natural birth of the litters, 2 pups/dam, for a total of 72 pups (40 males), were randomly selected according to a random number table and used for a water maze experiment. After the water maze experiment was complete, the offspring were euthanized by intraperitoneal injection of an overdose of 3% sodium pentobarbital (150 mg/kg). Then, the brain tissues were collected. The brain tissues of 6 pups per group were fixed in 4% paraformaldehyde for 24 h. The brain tissues of another 6 pups per group were placed in liquid nitrogen for Western blot detection, and the brain tissues of 6 pups per group were stored at 4 °C for ELISA detection.

### Morris water maze experiment

A Morris water maze analysis system (XR-XM101, Xmaze™) was used for the experiment. A circular swimming pool (diameter 160 cm, depth 40 cm) was filled with water at a temperature of 23 ± 1 °C, and an underwater platform (diameter 10 cm, placed 2 cm below the water surface) was placed in the first quadrant of this swimming pool. On the 30^th^ postnatal day, the pups were subjected to training. They were placed in the water at a designated starting point. If the pups found the platform within 90 s and stayed on it for 15 s, the trial ended. If the young rats failed to find the platform within 90 s, they were placed on the platform and allowed to stay for 15 s. The swimming time and speed of the pups were recorded. The above experiment was carried out 4 times a day for 5 days, starting at 9 o’clock every morning. After the training experiment was complete, the platform was removed, and the probe experiment was carried out [16].

### ELISA

The hippocampus was centrifuged (800* g*) for 15 min at 4 °C, and then the supernatant was collected. ELISA kits were utilized according to the kit instructions to detect the expression of IL-1β (Z02978-1, Genscript, Guangzhou, China), TNF-α (Z02999-10, Genscript), and IL-6 (CG39, Novoprotein, Guangzhou, China) in the supernatant.

### Nissl staining

The brain tissues of the offspring rats were fixed in a 4% paraformaldehyde solution and embedded in paraffin. The tissues were sectioned to a thickness of 5 μm. Then, these paraffin-embedded sections were dewaxed using xylene and rehydrated with gradient ethanol. After being washed, the sections were stained with 1% cresyl violet (G1430, Solarbio, Beijing, China) for 30 min. The sections were washed with distilled water again and treated with 70% alcohol for 60 s. Following dehydration with 70% ethanol, 80% ethanol, and 95% ethanol for 2 min each, the sections were treated with 100% ethanol for 5 min and xylene twice for 10 min each. Finally, a neutral gum sealant was used to seal the sections. The results were observed under a fluorescence light microscope (Revolbe FL, Echolaboratories, USA). The Nissl bodies fluoresced purple, and the nuclei fluoresced light purple.

### Immunohistochemistry

Using the same method as above, the brain tissue of young rats was fixed, embedded, sliced, dewaxed, and rehydrated. After treatment with 3% H_2_O_2_ methanol solution for 12 min, the slices were immersed in citrate buffer (pH 6.0, M053201, Mreda, Beijing, China) at 95 °C for 10 min and cooled. Subsequently, they were blocked with bovine serum albumin (5% BSA, A8010, Solarbio, Beijing, China) for 20 min. Polyclonal primary antibodies, comprising rabbit anti-human BDNF antibody (1:300, orb10181, Biorbyt, Wuhan, China) and rabbit anti-human CPEB2 antibody (1:300, orb182706, Biorbyt, Wuhan, China), were added and reacted for one night at 4 °C. After being washed with PBS 3 times for 5 min each, the sections were incubated at room temperature with Alexa Fluor 546 goat anti-rabbit IgG secondary antibody (1:1000, A-11010, Thermo Fisher, Shanghai, China) diluted with PBS. Then, hematoxylin stain was applied for 2 min, and alcohol hydrochloride was applied for 2 s for differentiation. Using the same method as above, the slices were dehydrated with ethanol, treated with xylene, sealed with neutral gum, and observed under a light microscope (Revolbe FL, Echolaboratories, USA). A corresponding area was selected on each slice and analyzed with ImageJ (version 5; National Institutes of Health) under the same conditions.

### Western blot

The hippocampus was lysed for 15 min on ice in RIPA buffer (R0010, Solarbio) containing protease and phosphatase inhibitors and subsequently centrifuged for 25 min at 12000* g*. A total protein extraction kit (BC3640-50 T; Solarbio) was used to obtain the total protein. 30 μl of protein was separated by 12% SDS–PAGE (Bio-Rad Laboratories, Inc.), transferred to a PVDF membrane (EMD Millipore), and finally blocked with 5% skim milk for 1 h. BSA (5%) was used to dilute the primary antibodies, and the sections were incubated with these antibodies overnight at 4 °C. Next, the following polyclonal primary antibodies were applied: rabbit anti-human NR4A1 antibody (1:1000, orb127604, Biorbyt, Beijing, China), rabbit anti-human NF-κB p-p65 antibody (1:100, orb501839, Biorbyt, Beijing, China), rabbit anti-human NF-κB p65 antibody (1:100, orb344389, Biorbyt), rabbit anti-human IκBα antibody (1:1000, 4812, Cell Signaling Technology, Shanghai, China), rabbit anti-human p-IκBα antibody (1:800, 2859, Cell Signaling Technology, Shanghai, China), and rabbit anti-human GAPDH antibody (1:1000, 5174, Cell Signaling Technology, Shanghai, China). Subsequently, the membrane was rinsed 3 times with TBS—0.01% Tween 20 (TBST) for 10 min each, incubated for 60 min at room temperature with Alexa Fluor 546 goat anti-rabbit IgG secondary antibody (1:1000, A-11010, Thermo Fisher, Shanghai, China) conjugated with horseradish peroxidase, and washed again. Finally, an enhanced chemiluminescence (ECL) reagent (GE2301, Genview, Shanghai, China) was used to observe the protein bands. ImageJ software (version 5) was used to quantify protein expression.

### Statistical analysis

SPSS 19.0 statistical analysis software (IBM, Chicago, IL, USA) was implemented to process the data, and all results are expressed as the mean ± standard deviation ($\overline{X}$ ± SD). One-way analysis of variance was exerted for data analysis among multiple groups, followed by Tukey’s test for post hoc analysis. *p* < 0.05 indicated that the difference was statistically significant.

## Results

### Effect of sevoflurane exposure during pregnancy on the memory and learning ability of offspring rats

**Fig. 2:**
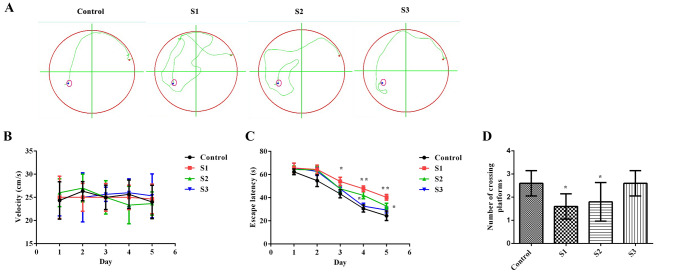
The Morris water maze test was used to measure the offspring rats’ behavior. **A** Swimming trajectory of a rat in each group on the third training day; **B** Swimming speed; **C** Escape latency; **D** Number of platform crossings in probe trial. *p < 0.05, **p < 0.01 compared to the control group. There were 18 rats in each group, and the data are presented as $\overline{X}$ ± SD. Control: control group; S1: sevoflurane exposure in early pregnancy; S2: sevoflurane exposure in middle pregnancy; S3: sevoflurane exposure in late pregnancy

To study the effects of sevoflurane exposure at different stages of pregnancy on the learning and memory of offspring rats, a water maze experiment was used to analyze the behavior of the offspring. Figure 2A represents the swimming trajectory of one rat in each group on the third training day. The S1 and S2 groups needed more time than the control and S3 groups to find the platform (Fig. 2A). Subsequently, the swimming speed (Fig. 2B), escape latency (Fig. 2C) and number of platform crossings (Fig. 2D) in the rat offspring were analyzed. The results showed that there were no obvious differences in swimming speed among these groups (*p* > 0.05). Compared with the results in the control group, the escape latency of the S1 group began to increase overtly on the 3rd training day (*p* < 0.05), the escape latency of the S2 group began to increase markedly on the 4th training day (*p* < 0.05), and the numbers of platform crossings in the S1 and S2 groups were significantly reduced (*p* < 0.05). These results demonstrated that sevoflurane exposure during early and middle pregnancy could substantially impair the later memory and learning ability of the offspring.

### Effects of sevoflurane exposure during pregnancy on inflammatory factors and Nissl bodies in the hippocampi of offspring

**Fig. 3:**
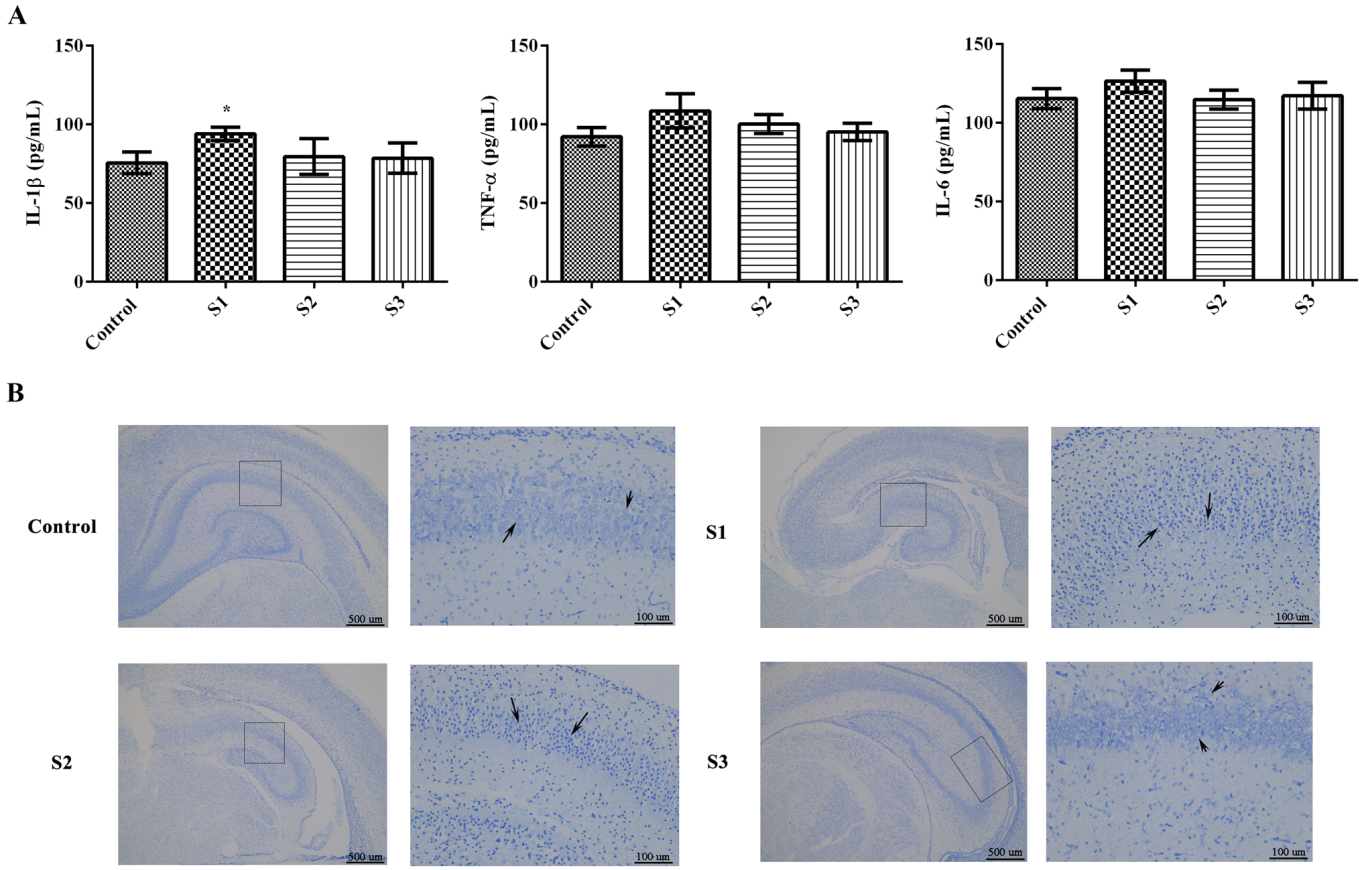
Sevoflurane anesthesia during pregnancy affected inflammatory factors and Nissl bodies in the hippocampi of the offspring. **A** ELISA was utilized to detect the expression of IL-1β, TNF-α and IL-6 in the brain tissues of each group; **B** Nissl staining was used to detect Nissl bodies in the hippocampus of each group. The black arrows represent Nissl bodies. There were 6 rats in each group, and the data are represented by $\overline{X}$ ± SD. *p < 0.05 compared to the control group. The magnification is 40 × in the first set of images and 200 × in the second set

The hippocampal expression of IL-1β, TNF-α, and IL-6 was assessed by ELISA (Fig. 3A). Compared to the control group, the S1 group had a marked increase in IL-1β (*p* < 0.05); however, the expression of TNF-α and IL-6 showed no significant differences among the groups (*p* > 0.05). Furthermore, Nissl staining was used to detect Nissl bodies in the hippocampi of offspring (Fig. 3B). The hippocampal structure of the control rats was intact, and the Nissl bodies were clearly defined. The S1 and S2 groups had sparse Nissl bodies compared with the control group. The hippocampi of the offspring in the S3 group were intact, and the Nissl bodies were clearly defined. These results suggest that sevoflurane anesthesia during early and middle pregnancy could significantly affect the formation of Nissl bodies in the hippocampi of offspring rats.

### Effect of sevoflurane exposure during pregnancy on BDNF and CPEB2 in the hippocampi of offspring rats

**Fig. 4:**
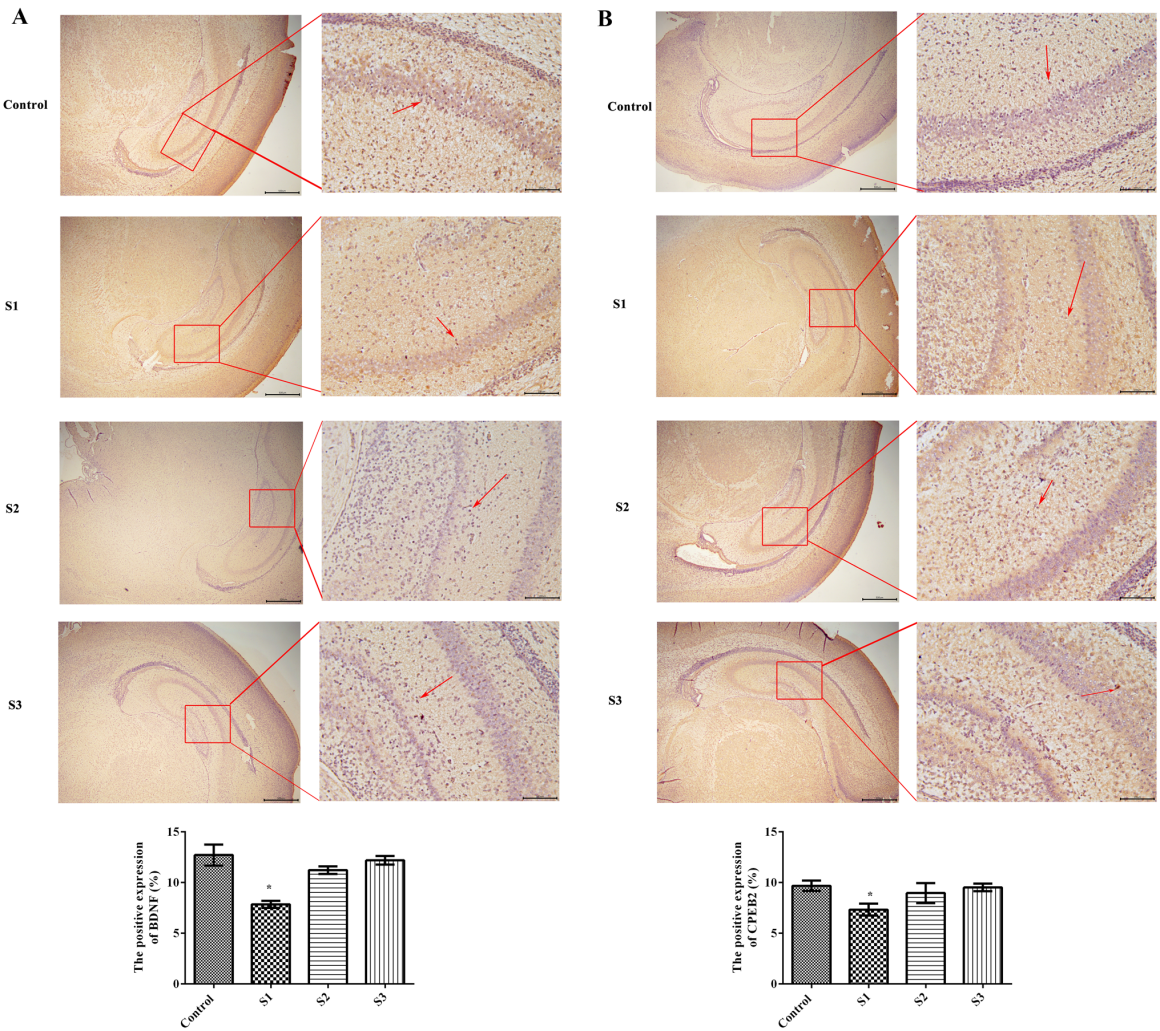
Effect of gestational sevoflurane anesthesia on BDNF and CPEB2 in the hippocampi of offspring rats. **A** BDNF expression was detected by immunohistochemistry; **B** CPEB2 expression was detected by immunohistochemistry. The magnification is 40 × in the first set of images and 200 × in the second set. The red arrow represents positive expression. There were 6 rats in each group, and the data are expressed as $\overline{X}$ ± SD

The expression levels of BDNF (Fig. 4A) and CPEB2 (Fig. 4B) in the hippocampus of offspring were analyzed by immunohistochemistry. Compared with the control condition, general anesthesia with sevoflurane in early pregnancy significantly reduced the expression of BDNF and CPEB2 in the hippocampi of the offspring (*p* < 0.05), which suggests that anesthesia in early pregnancy could significantly affect the expression of BDNF and CPEB2 in the hippocampi of offspring rats.

### Effect of sevoflurane exposure during pregnancy on the NR4A1/NF-κB pathway in the hippocampi of offspring

**Fig. 5:**
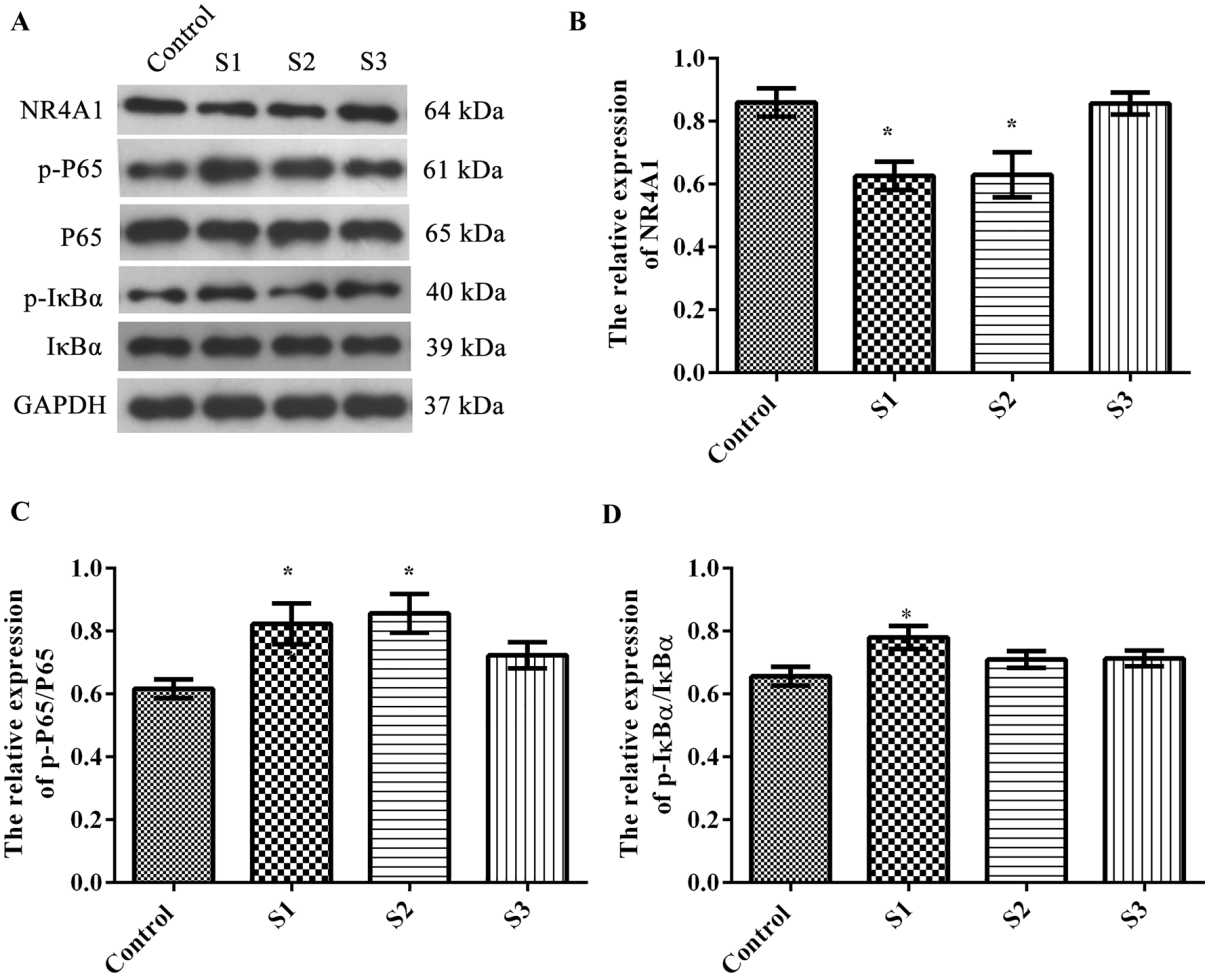
Effect of sevoflurane anesthesia during pregnancy on proteins related to the NR4A1/NF-κB pathway in the hippocampi of offspring rats. **A** Western blot analysis of NR4A1, p-P65, P65, p-IκBα, and IκBα protein; **B** Relative expression of NR4A1; **C** Relative expression of p-P65/P66; **D** Relative expression of p-IκBα/IκBα. There were 6 rats in each group, and the data are represented as $\overline{X}$ ± SD. *p < 0.05 compared to the control group

Western blotting (Fig. 5A) was used to analyze the expression of nuclear receptor subfamily 4 (group A-1, NR4A1), p-P65/P65 and p-IκBα/IκBα in the hippocampi of offspring. In the S1 and S2 groups, the expression levels of NR4A1 were lower than that in the control group (Fig. 5B *p* < 0.05), and the phosphorylation ratio of P65 was higher than that in the control group (Fig. 5C* p* < 0.05). The phosphorylation ratio of IκBα in the S1 group was significantly higher than that in the control group (Fig. 5D *p* < 0.05). This suggests that anesthesia in early pregnancy can significantly affect the expression of proteins related to the NR4A1/NF-κB pathway in the hippocampi of offspring rats.

## Discussion

Sevoflurane is often used for anesthesia during pregnancy, and many studies have begun to preliminarily explore whether and how sevoflurane may have lasting harmful effects on the brains of gestationally exposed offspring. Multiple mechanisms are involved in neuronal damage to offspring, for example neuronal apoptosis [16], synaptic plasticity [17], and BDNF-related pathways [18, 19]. Chai et al. [20] reviewed the fundamentals of fetal toxicity related to gestational sevoflurane exposure and demonstrated that the pathology associated with fetal toxicity involves oxidative stress, neuroinflammation, neuronal apoptosis, and alteration of synaptic properties. However, existing studies have mainly focused on the effects of sevoflurane exposure in a single stage of pregnancy [16–19]. In this paper, we compared the effects of sevoflurane exposure during early, middle and late pregnancy on the learning and memory of rat offspring as determined by a water maze test. The results showed that sevoflurane exposure in early and middle pregnancy could damage the learning and memory capacity of rat offspring. However, sevoflurane exposure during late pregnancy did not affect the learning and memory ability of the offspring. These results were consistent with previous studies [17, 19, 21].

In this study, we evaluated the changes in Nissl bodies and in hippocampal BDNF and CPEB2 expression in the offspring to identify possible reasons for the decline in learning and memory ability. BDNF plays a key role in brain development and function; its expression is driven by histone acetylation and depends on age in humans and rodents [22]. Signaling cascades activated by BDNF and its receptor, the receptor tyrosine kinase TrkB, link neuronal growth and differentiation with synaptic plasticity [23]. CPEB2 also plays an important role in learning and memory by contributing to synaptic plasticity. In contextual fear conditioning and Morris water maze tests, forebrain-restricted CPEB2 conditional knockout mice exhibited impaired hippocampus-dependent memory [24]. In this study, we found that Nissl bodies were dissolved in the hippocampi of offspring rats when the mothers were exposed to sevoflurane during early and middle pregnancy, and the levels of BDNF and CPEB2 in the hippocampus of offspring rats were obviously decreased by maternal sevoflurane exposure during early pregnancy. These outcomes suggested that sevoflurane exposure in early pregnancy damages the neuronal growth of rat offspring.

In this article, we also found that the levels of inflammatory cytokine IL-1β in the hippocampus of offspring were increased during sevoflurane exposure in early pregnancy, suggesting that neuronal damage after sevoflurane exposure is related to the activation of inflammation-related pathways. Then, NR4A1, p-p65/p65, and p-IκBα/IκBα expression were analyzed in the hippocampi of offspring. Interestingly, we found that NR4A1 expression was significantly decreased but the expression of p-p65/p65 and p-IκBα/IκBα was markedly increased in the hippocampi of rat offspring when the mothers were treated with sevoflurane during early and middle pregnancy. NR4A1 is a member of the NR4A family, which is associated with cellular processes [25]. Jiang et al. found that pretreatment with the NR4A1 activator cytosporone B blocked endothelin-1 expression in the lungs of LPS-exposed rats, and this suppression was mediated by NF-κB signaling [26]. In particular, NR4A1 can upregulate the expression of downstream targets of p65 by weakening the binding ability of p65 and DNA; directly promoting the expression of other NF-κB inhibitors, such as IκB [27]; and indirectly regulating the activity of the NF-κB pathway through non-protein–protein interactions [28]. In agreement with these previous studies, this study demonstrated that sevoflurane exposure during early pregnancy in rats damages the neuronal growth of the offspring by affecting the NR4A1/NF-κB pathway to increase the hippocampal secretion of inflammatory cytokines.

Most general anesthetics are lipophilic and easily cross the placenta, and neurogenesis, neuronal migration, and corticogenesis are major neurodevelopmental events in early and middle pregnancy [29], which may explain why the neurotoxic effect of anesthesia on the brain development of offspring is greater in early gestation than in late gestation. The long-term neuroinflammatory effect of sevoflurane may be mediated by the release of inflammatory mediators, which would further aggravate the oxidative stress response [20]. These mediators of neuroinflammation could induce neurobehavioral dysfunction, and cause cognitive dysfunction. Cellular damage due to the disruption of membrane lipids, acid esters, and lipoproteins induces neurobehavioral dysfunction and causes cognitive dysfunction. In addition, sevoflurane-induced synaptic dysfunction is a downstream consequence of tau phosphorylation, and tau associated with neuroinflammation and synapse dysfunction [30]. However, there still exist many limitations in our research. For example, there is no direct evidence that NR4A1, p65, and IκBα are regulated by sevoflurane in the same pathway during early pregnancy, and the effect of NR4A1 downregulation on the expression of BDNF and CPEB2 during pregnancy is unclear. The relationship between NR4A1 and tau is also needed to explore. Therefore, additional experimental and mechanistic studies are required to identify the expression of memory-related genes during learning and memory formation, as well as their involvement in memory impairment induced by maternal sevoflurane exposure during pregnancy.

## Conclusion

In rats, sevoflurane anesthesia has a greater impact on the learning and memory of offspring when administered during early or middle pregnancy than during late pregnancy, and the mechanism of this effect might be associated with the NR4A1/NF-κB pathway.
